# Expenditures of medicine use in hypertensive/diabetic elderly and physical activity and engagement in walking: cross secctional analysis of SABE Survey

**DOI:** 10.1186/s12877-017-0437-0

**Published:** 2017-03-20

**Authors:** Denise Rodrigues Bueno, Maria de Fátima Nunes Marucci, Luis Alberto Gobbo, Manuela de Almeida-Roediger, Yeda Aparecida de Oliveira Duarte, Maria Lucia Lebrão

**Affiliations:** 1 Department of Nutrition, Faculty of Public Health, University of Sao Paulo, Avenida Doutor Arnaldo, 715, Cerqueira César, São Paulo, SP CEP: 01246-904 Brazil; 2 Department of Nutrition of Faculty of Public Health, University of São Paulo, São Paulo, Brazil; 3 Department of Physical Education, Univ. Estadual Paulista, Presidente Prudente, SP Brazil; 4 Department of Nursing of School of Nursing, University of São Paulo, São Paulo, Brazil; 5 Department of Epidemiology of Faculty of Public Health, University of São Paulo, São Paulo, Brazil

**Keywords:** Expenditures, Physical Activity, Elderly, Medicine use, Hypertension, Diabetes

## Abstract

**Background:**

The literature shows the inverse association between physical activity level (PAL) and chronic diseases that have a significant burden over health care costs. However, in upper-middle income countries and in elderly population this information are scarce.

**Objective:**

To describe the annual drug expenditures for the hypertensive and diabetic elderly population in Brazil and to analyze the association with PAL and engagement in walking.

**Methods:**

This cross sectional study is part of SABE Survey and comprised 806 hypertensive and/or diabetic elderly (≥60 years old). The annual expenditures of medicine use was estimated for all medications for hypertension and/or diabetes they were taking. The PAL was considered insufficient when moderate physical activity was <150 min/week or vigorous physical activity was < 75 min/week. Engagement in walking was considered by at least 1 day a week. All expenditures were presented through the descriptive values (in American Dollars US$) according PAL and engagement in walking. The association analysis between annual expenditures, PAL and engagement in walking were performed by multiple logistic regression models adjusted for gender, age and body mass index.

**Results:**

The average annual cost was higher in diabetic and insufficient physically activity elderly. The 1-year estimated.cost was US$ 73386,09 and 295% higher in insufficiently physically active. Older people who reported not walking had a higher risk to higher annual expenditures of medicine use (OR = 1.57, 95% CI 1.03–2.40).

**Conclusions:**

The annual expenditures of medicine use for controlling hypertension and diabetes of Brazilian elderly were higher and inversely associated with physical activity level and engagement in walking.

## Background

The prevalence of hypertension (HTS) among elderly latinos (Mexico, Paraguay and Venezuela) is 67.3% in men and 61.1% in women, increasing to 70% among those seniors aged 70 or higher [1]. For diabetes mellitus (DM), according to data provided by the Base Population Health Census taken in municipalities in São Paulo state, the disease was self-reported by 14.9% of men and 15.8% of women over 60 years old [2].

There is increasing literature, identifying that chronic diseases have a significant burden over health care costs [3, 4]. In the pharmacological intervention for control of HTS and DM, the simultaneous use of more than one type of drug is quite common, with direct consequences to the costs billed to the health system. Compared to normotensive and non-diabetic patients, drug costs are higher for those affected by the disease [5, 6] Among the non-pharmacological methods of control, physical activity (PA) is widely recommended for hypertensive [7] and diabetic persons [8] due to its effect in lowering at-rest blood pressure [9] and glycemia [10, 11]. Williams [12] showed that the use of drugs was lower as a result of engaging in walking exercise. Other studies showed that intervention by means of PA results in lower overall health care costs [13] and, specifically, lower drug use [14, 15]. Analysis of expenditures in Canadá estimates that in 2009 the direct costs due to hypertension (US$ 588 million) and DM (US$ 470 million) directly attributable to physical inactivity (<150 min per week) are higher than those among active people [16]. Tsuji et al. [17] demonstrated lower healthcare costs in a population of physically active seniors.

Investigations of this nature are extremely important given the phenomenon of the transition of the populational age structure, and the resulting increase in the numbers of older people, who also have the highest health care costs in comparison to young adults [6, 18]. A Brazilian study estimated that the costs of controlling DM will reach US$ 8.7 billion in 2050, corresponding to the increase of US$ 3.7 billion when compared to 2010. For HTS, the analyses indicate that, in 2050, the cost will reach US$ 657 million [19]. In the US, the cost of drugs for treatment of DM represents 28% of the available healthcare resources and for treatment of hypertension the main cost was the use of drugs [20].

However, information of this nature in Brazil is still scarce, especially as regards large populational studies focused on the elderly*.* The aim of this study was to describe the annual drug cost for the elderly population of the city of São Paulo and to analyze the association of these costs with PA level and walking.

## Methods

### The SABE Survey and sample

The SABE Survey (Health, Welfare and Aging Survey) is a home-focused epidemiological study and the aim was to investigate health characteristics relating to the elderly population in developing countries. In Brazil, the SABE Survey was developed in the urban area of the capital of São Paulo state and it was approved by the Ethics Committee of the Public Health School of University of São Paulo, control number 475,455. The participants were invited to read and approve a consentient letter before to start all the assessments and interviews, where was explained about sharing their data (age, self-reported data, physical activity level and others) to scientific work, and agreeing about their names or personal data wouldn’t be used in the publications. The datasets generated and/or analyzed during the current study which were used under license for the current study are not publicly available due to the policy of SABE Survey, and so are not publicly available. Data are however available from the authors upon reasonable request.

For this cross-sectional study, the SABE data used was collected in 2010, and comprised a total of 1,211 elderly people. However, in order to meet the objective of investigating the association of drug costs among hypertensive and/or diabetic seniors, only those who reported these diseases in the interview were included in this sample. Those seniors were also excluded that did not include complete information in the questionnaire, as was necessary for analysis (body mass or/and height). Having satisfied the requirements described, 806 elderly individuals participated in the study.

### Outcome variable - annual expenditures of medicine use

The elderly individuals were told to describe all medications they were taking, so that the analysis could include both prescription and self-medicating/over-the-counter drugs. Herbal medicines were not considered, given that they are not included among those provided by Unified Health System in Brazil. Those drugs that were bought from formularies were also not considered, given the impossibility of obtaining standard pricing. The exact nature of drug costs included only those drugs that were identified as being indicated for the treatment of HTS and DM and became apparent after three stages:

1. *Estimated daily amount consumed:* this was determined by the Defined Daily Dosage (DDD), which is the average daily dosage of the drug in its use for its main indication [21];
2. *Price identification:* this data was taken from a survey of the Healthcare Price Database (http://www.bps.saude.gov.br), and considered the amount of the dosage unit (1 tablet, 1 pill or 1 capsule).
3. *Estimation of monthly and annual expenditures:* the daily cost estimated from the DDD was multiplied by 30 (days) for each product, and then multiplied by 12 (months), to reflect the annual cost. Then, a currency value update was carried out based on the most recent data available from the Consumer Price Index as provided by the Brazilian Institute of Geography and Statistics (IBGE-IPCA).

Annual expenditures of medicine use was divided into quartiles, with the last quartile of cost (US$ ≥ 75th) considered as highest expenditures group. The first three quartiles (US$ < 75th) represent the lowest annual expenditures group.

### Explanatory variable

#### Physical activity level and walking

The participants’ physical activity was self-reported using the short version of the *International Physical Activity Questionnaire* (IPAQ). The elderly were classified according to their physical activity level (PAL) in accordance with current recommendations [22]: i. *Active:* ≥ 150 min/ week of moderate PA, or ≥ 75 min/ week of vigorous PA; ii. *Insufficiently active:* <150 min/ week of moderate PA or <75 min/ week of vigorous PA.

Walking was reported according to the weekly frequency of engagement. The elderly were divided into two groups: *i) Yes:* Seniors who walked at least 1 day a week; *ii) No:* older adults who did no walking. Moreover, to see the difference on costs according to the frequency of walking in a week they were divided into three groups: *i) ≥ 4 days a week; ii) 1 to 3 days per week; iii) does not walk* (0 days per week).

### Statistical analysis

This is a population-based study derived from complex samples. So, the statistical analyses and tests used were those indicated for use with *survey-type* studies (svy).

For the complete visualization of costs, in Dollars (US$), they are presented through the mean values and their respective 95% confidence intervals (CI 95%), median values and values of percentile 25 (25th) and percentile 75 (75th).

To association analysis the expenditures of medicine use (outcome variable) was divided into quartiles, with the last quartile of cost (US$ ≥ 75th) considered a risk category (highest cost). The first three quartiles (US$ < 75th) are representing the reference category (lowest cost). The multiple logistic regression models (Rao & Scott test) were adjusted for sex, age and body mass index, and comorbidities.

To examine associations of costs with PAL (reference: active, risk: insufficiently active) and walking (reference: walking ≥ 4 days, risk: walking < 4 days) by gender, three multiple logistic regression models were performed separately, using these variables as explanatory variables, including analysis for men and women. The overall regression model was performed adjusted by gender, Body Mass Index (BMI), age, marital status, socioeconomic status and education level. The other analyses (separated according to gender) were adjusted for age and BMI. The significance level was established in 5% for all analysis and statistical analyses were provided by Stata 10.1.

## Results

Of the 806 elderly individuals in the sample, 60.9% were female (*n* = 526), 95.9% (*n* = 767) reported being affected by high blood pressure and 62.5% (*n* = 295) for diabetes, with no differences in the prevalence of HTS and DM by gender (p > 0.05). The average age of the sample was 70.7 (95%-CI = 70.2 to 71.3) years of age and does not differed between the hypertensive and diabetic.

**Fig. 1:**
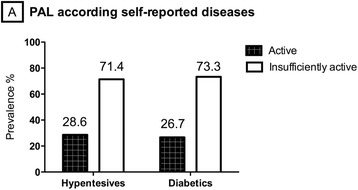
Prevalence of inactive and active individuals according to the self-reported chronic disease

According to the self-reported weekly time of physical activity, 72% (*n* = 575) of the elderly were classified as insufficiently active and of these, 54.7% were women. 39.4% of the sample self-reported the engagement in walking in ≥ 4 days/week (48.7% men and 33.4% women, *p* = 0.002) and 32% self-reported no walking (no difference between sex). The chi-square test demonstrated no difference in the PAL with the prevalence of hypertension and diabetes (*p* = 0.14 and *p* = 0.55, respectively). As can be seen from the results shown in the graph in Fig. 1, the majority of the elderly, both hypertensive (71.4%) and diabetic (73.3%) were classified as insufficiently active.

The total number of drugs for hypertension and diabetes was 2,141, being 1,770 antihypertensive and 371 anti-diabetic, with an average of 2.6 (95%-CI 2.5–2.7) drugs per elderly, with no difference between sexes (p > 0.05). In seniors who only had HTS, the average was 2.0 (95%-CI 1.9–2.2) medicines without a difference according PAL (p > 0.05). The average use of antidiabetic drugs in patients that reported only DM was 2.2 (95%-CI 1.7–2.7), with no difference between PAL and walking exercise (p > 0.05). In patients that reported both diseases, the average of antihypertensive and antidiabetic drugs was 3.8 (95%-CI 3.6–4.1). When considering all the drugs taken by the 806 seniors, 72.2% were presented by elderly people with an insufficient PAL.

The average annual cost for drugs per individual was US$ 85.40 (IC95% 68.79–102.00), similar by sex and higher in the groups of more elderly people with insufficient PAL. The 1-year estimated cost was US$ 73.386,09 for the 806 older adults in this sample (Table 1), and 295% higher in the group classified as being insufficiently physically active. Analyzing costs by the median, the costs were higher for the same groups when compared to the average; albeit with less of a difference between the values.

**Table 1:** Description of annual expenditures for medicine use according gender, PAL and walking. (São Paulo, 2015 [*n* = 806])

	Annual expenditures of medicine use-US$	
Variable	Mean (CI 95%)-Median [25^th^- 75^th^]	Sum - ∑
All sample	85.40 (68.79–102.00) – 20.89 [15.05; 53.05]	73.386.09
Gender		
Men	84.90 (54.55–115.25) – 17.71 [3.22; 49.03]	25.553.35
Woman	85.72 (66.65–14.79) – 21.69 [4.82; 54.64]	47.832.74
PAL		
Active	60.57 (43.83–77.32) – 16.67 [4.82; 43.42]	14.816.44
Insufficiently active	95.07 (72.99–117.16) – 22.49 [4.82; 54.68]	58.569.66
Walking		
Yes	77.55 (59.57–95.54) – 17.71 [3.22; 44.97]	42.918.59
No	102.03 (67.05–137.02) – 25.71 [6.41; 62.64]	30.467.51
Walking		
No Walking	102.03 (52.16–107.92) – 25.71 [6.41; 62.64]	30.467.51
1–3 days	74.13 (55.32–92.94) – 20.89 [4.82; 57.87]	16.534.61
≥ 4 days	80.04 (67.19–137.02) – 17.67 [3.22; 41.79]	26.383.96

*Note*: *PAL* Physical activity level, CI 95%– confidence interval 95%

### Costs by medication type

The medicines acting on the renin-angiotensin system were higher (24%), but its total cost in 1 year was lower than that observed in the use of antidiabetic, antihypertensive, as well as lipid-lowering, agents, which amounted to the highest annual cost (Table 2).

**Table 2:** Prevalence of medicine use (%) and annual expenditures sum (∑ − US$) according to medicine type. (São Paulo, 2015 [*n* = 806])

Type of medication	Prevalence of medicine use (%)	∑ Annual Cost US$
Renin Angiotensin System controller	24.3	4782.19
Diuretics	18.4	3155.22
Anti-diabetics	17.8	13244.32
Lipid-lowering agents	11.5	20221.64
Beta Blockers	10.7	4141.92
Calcium channel blockers	10.2	1622.26
Cardiovasculars	4.1	12710.62
Anti-hypertensive	2.0	10667.27
Vasodilators	1.0	745.99

The multiple association analysis that was performed to all sample demonstrated that not walking was directly associated with risk to higher annual spending with medicine use, adjusted for confounders. Male group had higher chances of high drug costs when they were classified as insufficiently active (OR 3.19, 95% CI 95% 1:10 to 9:30). Women who did not report walking were more likely to have higher annual medicine costs (OR 1.83, 95% CI 95% 1:08 to 3:09) (Table 3).

**Table 3:** Association between PAL and walking with higher annual expenditures of medicine use (≥75th), to overall sample and according to gender

	Annual expenditures of medicine use (≥75^th^)								
	Overall (*n* = 806)			Men (*n* = 281)			Women (n = 525)		
Groups/Variable	OR	CI 95%	p	OR	CI 95%	*p*	OR	CI 95%	*p*
Model 1 PAL									
Active	1.00	--		1.00	--		1.00	--	
Insuf. Active	1.27	0.86–1.86	0.23	3.19	1.10–9.30	0.03	0.94	0.41–2.20	0.90
Model 2 Walking									
Yes	1.00	--		1.00	--		1.00	--	
No	1.39	0.96–2.00	0.07	1.04	0.53–2.02	0.91	1.51	0.97–2.35	0.07
Model 3 Walking									
≥ 4 days	1.00	--	--	1.00	--		1.00	--	
1–3 days	1.56	1.00–2.43	0.05	1.78	0.78–4.07	0.16	1.42	0.83–2.43	0.20
No Walking	1.70	1.11–2.58	0.01	1.27	0.62–2.60	0.51	1.83	1.08–3.09	0.02

*Note*: *PAL* Physical activity level, *OR* Odds ratio, *CI* Confidence interval, Logistic regression models adjusted for gender, *BMI* age, marital status, socioeconomic status, andeducational level

**Fig. 2:**
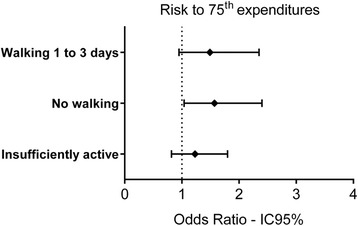
Odd s ratio to highest expenditures according to engagement in walking. Logistic regression models

When considering all the sample, regardless of gender and disease, the results presented in Fig. 2 show that there was no association of the overall PAL with highest annual cost of medication (OR = 1.21, 95% CI 95% 0.82–1.80), adjusted for gender, age and BMI. However, as it related to walking, and stratified by the frequency of their engagement in walking, older people who reported not walking during the week had a higher risk of being included in the group reporting higher medication costs (OR = 1.57, 95% CI 95% 1.03–2.40), compared to those seniors who reported walking on four or more days a week (Fig. 2).

## Discussion

The aim of the present study was to describe the annual medication expenditures for hypertensive and diabetic older adults and to analyze their associations with PAL and weekly engagement in walking. The results showed inverse associations for engagement in walking, adjusted for gender, age and BMI.

As indicated in the literature [5], hypertensive had higher use and cost of medications when compared to normotensive. Therefore, studies suggest that PA, especially aerobic training, helps in keeping blood pressure at normal levels, resulting in a reduced need for medication use and costs. Even though the PA was of moderate intensity, such as walking [22–24], it exerts hypotensive effects and controlling the disease. This study was grounded in this information and therefore was based on the assumption that costs would be lower among physically active hypertensive older adults. Indeed, our results are confirming the literature because indicates that there is a positive and direct relationship between the annual cost per person and the control rate of hypertension, where-in Brazil-the monthly cost for the pharmacological control of hypertension is lower when blood pressure was < 140/90 mmHg [25].

Likewise, the cost for non-diabetic elderly is lower than the cost for diabetic [26] and physical inactivity is present in most individuals affected by DM [27]. On the other hand, there are also indications that a physically active lifestyle mitigates this situation and contributes to the regulation of blood glucose [28, 29] and a decreased risk of complications related to DM due to the improved metabolic profile of these individuals [27]. Thus, in addition to the cost of the disease, diabetic individuals with a lower PAL present higher costs for medications for other conditions [30].

In direction of these findings, in a 2-year study of Italian diabetic sufferers with an average age of 62, those who managed to increase energy expenditure through PA showed an equivalent reduction in drug use costs [11]. But, these results about PAL were not concordant with our results. However, our findings are in agreement with Codogno et al. [31], who did not observe lower drug costs for these different PAL among diabetic elderly, however, there was an inverse association of costs with the overall PA (OR 2.12, 95% CI 1.18–3.78) when considering the domains of work and sports activities together. On the other hand, the costs for controlling both diseases were lower with engagement in walking as shown by Williams [12] who showed a lower rate of use of antidiabetic and antihypertensive medications in physically active individuals. Thus, further studies with the elderly population are needed to more clearly elucidate the associations of physical activity and walking to reducing drug costs.

On these disagreements in literature, a plausible explanation would be the self-reported method of analysis of physical activity. It is noteworthy that the PAL estimated by the IPAQ in this study was largely carried out in the free time of those who reported it. In addition, studies demonstrate that the PAL estimated by the IPAQ may be overestimated [32] to the extent that seniors classified as physically active may not actually be, thus diminishing the strength of the association of physical inactivity to costs for the entire sample. On the other hand, these results are similar to Tsuji et al. [16], who observed inverse associations between cost and engagement in walking. In this regard, IPAQ validation studies show that the correlation of responses relating to walking with objective measurements (accelerometer) is greater than that relating to the responses for moderate and vigorous PA, especially in older women [33]. Thus, different areas of PA can be linked in different ways to healthcare costs, and the sum of daily energy expenditure may be the real determinant in cost reductions, such that further studies are needed to investigate these associations in Brazilian older adults, the results of which would indicate the best alternatives for intervention projects.

As regards the expenditures in diabetics, one possible explanation is the need for constant use of insulin, regardless of the PAL due to dysfunction of beta cells, which irreversibly reduces their natural production. Kaizu et al. [10] observed that although leisurely PA entails positive effects on the metabolic profile of diabetic patients, the proportion of individuals using oral hypoglycemic agents was not lower in individuals who were classified as active.

As it relates to public health interventions, the results presented are of extreme value and, when added to the results of other studies [34, 35], demonstrated that non-pharmacological intervention procedures for blood glucose and blood pressure control, especially through engagement in walking, have conclusive results and are cost-effective, and point to possible cost savings through primary prevention strategies. Thus, it can be inferred that these intervention strategies can result in the positive effect of reducing the costs associated with the control of the disease, since the cost savings at this primary level would mean lower costs at subsequent levels of public health services [36].

The PA at internationally recommended levels [22, 37] was low for both hypertensive and diabetic subjects. It is worth noting that studies are consistent in showing a greater prevalence of hypertension in insufficiently active individuals, especially among older adults [38]. Additionally, there is evidence that the risk of hypertension decreases concomitantly as level of cardiorespiratory fitness is increased [40]. Thus, our results are showing that the primary prevention strategy for reducing risk variables through aerobic training as walking seems to be feasible and economically interesting. Specifically in the case of Brazilian costs, a study published in 2010 showed that the cost of HTS reached US$ 398.9 million, consuming 1.43% of the resources of the nationalized healthcare program [41], and of this amount, 52.3% was spent with medication. Thus, since walking can be an aid in controlling the disease and reducing medicine use, this study points to a necessary and plausible intervention strategy to be employed among the older adults, aimed at saving financial resources for healthcare program.

In addition, the benefits of PA or walking may be more significant if we consider the lower costs classified as indirect and/or intangible, both by improving quality of life and well-being, as well as the inverse association of PAL to the risk of mortality among the hypertensive [42] and diabetic [43]. Other Brazilian population studies should be performed with the purpose of presenting the indirect costs associated with physical inactivity in order to strengthen the basis for the elaboration of public policies aimed at saving financial resources.

This study stands out for having been conducted with a representative sample of older adults living in São Paulo, however, it has some limitations. Readers need to do generalizations carefully. There are not a rural sample in this analysis, and Brazilians older people are classified according to the age ≥ 60 years-old. Although the estimate of the PAL through the IPAQ is feasible in epidemiological studies [33] and commonly performed in population surveys [39, 44, 45], it can be overestimated [46], and merits care in the interpretation of the results presented. Another limitation is the self-reported diagnosis of HTS and DM via interview format, which increases the chance of healthy seniors composing the sample and/or hypertensive or diabetic seniors being excluded from the analysis. It is noteworthy that this study did not carry out comparisons between elderly patients affected and not affected by the disease, nor were HTS and DM considered the conclusion of the analysis. Thus, memory bias does not seem to be a problem for the interpretation of the results of this study.

## Conclusions

In conclusion, engagement in walking was inversely associated with the annual cost due to medication use, independent from body mass index, gender and age, among hypertensive and/or diabetic older adults living in São Paulo city, Brazil. The epidemiological evidence of this study conducted with a representative sample of older hypertensive and diabetic individuals indicates the prospect of physical activity interventions. Thus, PA plays an important task saving public health resources and controlling chronic diseases among the elderly adults. Moreover, the cross-sectional design prejudices the conclusions regarding the relationship between medicine use expenditures and PAL or walking.

## Abbreviations

BMI: Body Mass Index
DDD: Defined Daily Dosage
DM: Diabetes Melitus
HTS: Hypertension
PA: Physical Activity
PAL: Physical Activity level

## References

[1] Kearney PM, Whelton M, Reynolds K, Muntner P, Whelton PK, He J (2005). Global burden of hypertension-analysis of worldwide data. Lancet.

[2] Francisco PMSB, Belon AP, Barros MBDA, Carandina L, Alves MCGP, Goldbaum M, Cesar CLG (2010). Self-reported diabetes in the elderly: prevalence, associated factors, and control practices. Cad Saude Publica.

[3] Arredondo A, Zúñiga A, Parada I (2005). Health care costs and financial consequences of epidemiological changes in chronic diseases in Latin America: evidence from Mexico. Public Health.

[4] Bahia L, Coutinho ES, Barufaldi L, de Azevedo Abreu G, Malhão T, Ribeiro de Souza C, Araujo D (2012). The costs of overweight and obesity-related diseases in the Brazilian public health system: cross-sectional study. BMC Public Health.

[5] Wang G, Yan L, Ayala C, George MG, Fang J (2013). Hypertension-associated expenditures for medication among US Adults. Am J Hypertens.

[6] Yang W, Dall TM, Halder P, Gallo P, Kowal SL, Hogan PF, Petersen M (2013). Economic costs of diabetes in the U.S. in 2012. Diabetes Care.

[7] Mancia G, Fagard R, Narkiewicz K, Redon J, Zanchetti A, Böhm M, Christiaens T, Cifkova R, De Backer G, Dominiczak A, Galderisi M, Grobbee DE, Jaarsma T, Kirchhof P, Kjeldsen SE, Laurent S, Manolis AJ, Nilsson PM, Ruilope LM, Schmieder RE, Sirnes PA, Sleight P, Viigimaa M, Waeber B, Zannad F, Burnier M, Ambrosioni E, Caufield M, Coca A, Olsen MH (2013). ESH/ESC guidelines for the management of arterial hypertension: The Task Force for the management of arterial hypertension of the European Society of Hypertension (ESH) and of the European Society of Cardiology (ESC). Eur Heart J.

[8] Colberg SR, Sigal RJ, Fernhall B, Regensteiner JG, Blissmer BJ, Rubin RR, Chasan-Taber L, Albright AL, Braun B (2010). Exercise and type 2 diabetes: The American College of Sports Medicine and the American Diabetes Association: joint position statement. Diabetes Care.

[9] Kokkinos PF, Giannelou A, Manolis A, Pittaras A (2009). Physical activity in the prevention and management of high blood pressure. Hellenic J Cardiol.

[10] Kaizu S, Kishimoto H, Iwase M, Fujii H, Ohkuma T, Ide H, Jodai T, Kikuchi Y, Idewaki Y, Hirakawa Y, Nakamura U, Kitazono T (2014). Impact of leisure-time physical activity on glycemic control and cardiovascular risk factors in Japanese patients with type 2 diabetes mellitus: The Fukuoka Diabetes Registry. PLoS One.

[11] Di Loreto C, Fanelli C, Lucidi P, Murdolo G, De Cicco A, Parlanti N, Ranchelli A, Fatone C, Taglioni C, Santeusanio F, De Feo P (2005). Make Your Diabetic Patients Walk. Diabetes Care.

[12] Williams PT (2008). Reduced diabetic, hypertensive, and cholesterol medication use with walking. Med Sci Sports Exerc.

[13] Wang F, McDonald T, Reffitt B, Edington DW (2005). BMI, physical activity, and health care utilization/costs among Medicare retirees. Obes Res.

[14] Bielemann RM, Knuth G, Hallal P (2010). Physical activity and costs savings for chronic diseases to the Sistema Único De Saúde. Rev Bras de Ativ Fís e Saúde.

[15] Bertoldi AD, Hallal PC, Barros AJD (2006). Physical activity and medicine use: evidence from a population-based study. BMC Public Health.

[16] Janssen I (2012). Health care costs of physical inactivity in Canadian adults. Appl Physiol Nutr Metab.

[17] Tsuji I, Takahashi K, Nishino Y, Ohkubo T, Kuriyama S, Watanabe Y, Anzai Y, Tsubono Y, Hisamichi S (2003). Impact of walking upon medical care expenditure in Japan: The Ohsaki Cohort Study. Int J Epidemiol.

[18] Hogan P, Dall T, Nikolov P (2003). Economic costs of diabetes in the US in 2002. Diabetes Care.

[19] Rtveladze K, Marsh T, Webber L, Kilpi F, Levy D, Conde W, McPherson K, Brown M (2013). Health and economic burden of obesity in Brazil. PLoS One.

[20] Degli Esposti E, Berto P, Ruffo P, Buda S, Degli Esposti L, Sturani A (2001). The PANDORA project: results of the cost of illness analysis. J Hum Hypertens.

[21] WHO Collaborating Centre for Drug Statistics Methodology (2012). Guidelines for ATC classification and DDD assignment 2013.

[22] WHO (2010). Global recommendations on physical activity for health. Geneva World Heal Organ.

[23] Lee LL, Arthur A, Avis M (2007). Evaluating a community-based walking intervention for hypertensive older people in Taiwan: A randomized controlled trial. Prev Med (Baltim).

[24] Lee LL, Watson MC, Mulvaney CA, Tsai CC, Lo SF (2010). The effect of walking intervention on blood pressure control: A systematic review. Int J Nurs Stud.

[25] Pardell H, Tresserras R, Armario P, del Rey RH (2000). Pharmacoeconomic considerations in the management of hypertension. Drugs.

[26] Hu R, Shi L, Pierre G, Zhu J, Lee D (2015). Diabetes and medical expenditures among non-institutionalized U.S.adults. Diabetes Res Clin Pract.

[27] Hermann G, Herbst A, Schütt M, Kempe H-P, Krakow D, Müller-Korbsch M, Holl RW (2014). Association of physical activity with glycaemic control and cardiovascular risk profile in 65 666 people with Type 2 diabetes from Germany and Austria. Diabet Med.

[28] Sigal RJ, Kenny GP, Boule NG, Wells GA, Prud D, Fortier M, Reid RD, Tulloch H, Coyle D, Phillips P, Jennings A, Jaffey J (2007). Annals of internal medicine article effects of aerobic training, resistance training, or both on glycemic control in type 2 diabetes.

[29] Bacchi PE, Moghetti NC, Zanolin ME, Milanese C, Faccioli N, Trombetta M, Zoppini G, Cevese A, Bonadonna RC, Schena F, Bonora E, Lanza M (2012). Metabolic effects of aerobic training. Diabetes Care.

[30] Codogno JS, Fernandes RA, Monteiro HL (2012). Physical activity and healthcares cost of type 2 diabetic patients seen at basic units of healthcare. Arq Bras Endocrinol Metab.

[31] Codogno JS, Turi BC, Kemper HCG, Fernandes RA, Christofaro DGD, Monteiro HL (2015). Physical inactivity of adults and 1-year health care expenditures in Brazil. Int J Public Health.

[32] Heesch KC, van Uffelen JG, Hill RL, Brown WJ (2010). What do IPAQ questions mean to older adults? Lessons from cognitive interviews. Int J Behav Nutr Phys Act.

[33] Tomioka K, Iwamoto J, Saeki K, Okamoto N (2011). Reliability and validity of the International Physical Activity Questionnaire (IPAQ) in elderly adults: the Fujiwara-kyo Study. J Epidemiol.

[34] Li R, Zhang P, Barker LE, Chowdhury FM, Zhang X (2010). Cost-effectiveness of interventions to prevent and control diabetes mellitus: A systematic review. Diabetes Care.

[35] Gillett M, Royle P, Snaith A, Scotland G, Poobalan A, Imamura M, Black C, Boroujerdi M, Jick S, Wyness L, Mcnamee P, Brennan A, Waugh N (2012). Non-pharmacological interventions to reduce the risk of diabetes in people with impaired glucose regulation: A systematic review and economic evaluation. Health Technol Assess (Rockv).

[36] Saha S, Carlsson KS, Gerdtham U-G, Eriksson MK, Hagberg L, Eliasson M, Johansson P (2013). Are lifestyle interventions in primary care cost-effective?-An analysis based on a Markov model, differences-in-differences approach and the Swedish Björknäs study. PLoS One.

[37] Nelson ME, Rejeski WJ, Blair SN, Duncan PW, Judge JO, King AC, Macera CA, Castaneda-Sceppa C (2007). Physical activity and public health in older adults: Recommendation from the American College of Sports Medicine and the American Heart Association. Med Sci Sports Exerc.

[38] Zaitune MPDA, Barros MBDA, César CLG, Carandina L, Goldbaum M, Alves MCGP (2010). Fatores associados à prática de atividade física global e de lazer em idosos: Inquérito de Saúde no Estado de São Paulo (ISA-SP), Brasil. Cad Saude Publica.

[39] Faselis C, Doumas M, Kokkinos JP, Panagiotakos D, Kheirbek R, Sheriff HM, Hare K, Papademetriou V, Fletcher R, Kokkinos P (2012). Exercise capacity and progression from prehypertension to hypertension. Hypertension.

[40] Rankinen T, Church TS, Rice T, Bouchard C, Blair SN (2007). Cardiorespiratory fitness, BMI, and risk of hypertension: The HYPGENE study. Med Sci Sports Exerc.

[41] Dib MW, Riera R, Ferraz MB (2010). Estimated annual cost of arterial hypertension treatment in Brazil. Rev Panam Salud Publica.

[42] Brown RE, Riddell MC, Macpherson AK, Canning KL, Kuk JL (2013). The joint association of physical activity, blood-pressure control, and pharmacologic treatment of hypertension for all-cause mortality risk. Am J Hypertens.

[43] Zethelius B, Gudbjörnsdottir S, Eliasson B, Eeg-Olofsson K, Cederholm J (2014). Level of physical activity associated with risk of cardiovascular diseases and mortality in patients with type-2 diabetes: report from the Swedish National Diabetes Register. Eur J Prev Cardiol.

[44] Vagetti GC, Barbosa Filho VC, Moreira NB, de Oliveira V, Mazzardo O, de Campos W (2013). The prevalence and correlates of meeting the current physical activity for health guidelines in older people: A cross-sectional study in Brazilian women. Arch Gerontol Geriatr.

[45] Bergman P, Grjibovski AM, Hagströmer M, Bauman A, Sjöström M (2008). Adherence to physical activity recommendations and the influence of socio-demographic correlates - a population-based cross-sectional study. BMC Public Health.

[46] Inoue S, Sugiyama T, Takamiya T, Oka K, Owen N, Shimomitsu T (2012). Television Viewing Time is Associated with Overweight/Obesity Among Older Adults, Independent of Meeting Physical Activity and Health Guidelines. J Epidemiol.

